# Should “medical students` disease” be regarded as a true disease entity? Cross-sectional study among Polish students

**DOI:** 10.1192/j.eurpsy.2022.1529

**Published:** 2022-09-01

**Authors:** K. Szczurek, N. Furgał, D. Szczepanek, K. Krysta, M. Krzystanek

**Affiliations:** 1 Medical University of Silesia, Students` Scientific Association, Department Of Rehabilitation Psychiatry, Katowice, Poland; 2 Medical University of Silesia, Department Of Rehabilitation Psychiatry, Katowice, Poland

**Keywords:** nosophobia, hypochondria, medical students

## Abstract

**Introduction:**

There is a widely known stereotype about medical majors repeated by generations of medical practitioners called ,,medical student disease”. It’s based on a belief that unexperienced students are prone to develop pathological fear of medical conditions they are studying about.

**Objectives:**

The aim of the study was to examine two populations of students - medical and non-medical ones in order to compare their level of hypochondriacal behavior and health-related anxiety. Moreover we looked for other factors which might have had an influence on hypochondria and nosophobia among them.

**Methods:**

The proprietary questionnaire was completed by 606 students (303 medical students of the Medical University of Silesia in Katowice and 293 students of the 3 largest non-medical universities in Katowice).

**Results:**

The results show that medical students receive same scores on a nosophobia scale as students of non-medical universities (p=0,5). The analysis of hypochondriacal behavior showed significantly higher results in non-medical students group (p=0,02) .The higher medical students were at the stages of academic education, the higher the results of nosophobia they obtained. In the entire study group female received higher score in relation to the fear of illness (p = 0.001). People with mental disorders achieve significantly higher results of nosophobia (p <0.001 in the entire group) and of hypochondria (p <0.001 for the entire cohort).

**Conclusions:**

Our study challenges the widespread belief that medical students, compared to their peers, are overly anxious about their own health. Gender and having a mental illness are predictors of hypochondria and nosophobia.

**Disclosure:**

No significant relationships.

